# Oculomotor Patterns in Children with Poor Reading Abilities Measured Using the Development Eye Movement Test

**DOI:** 10.3390/jcm13154415

**Published:** 2024-07-28

**Authors:** Danjela Ibrahimi, Marcos Aviles, Juvenal Rodríguez-Reséndiz

**Affiliations:** 1 Facultad de Medicina, Universidad Autónoma de Querétaro, Santiago de Querétaro 76010, Mexico; danjela.ibrahimi@uaq.mx; 2 Facultad de Ingeniería, Universidad Autónoma de Querétaro, Santiago de Querétaro 76010, Mexico; juvenal@uaq.edu.mx

**Keywords:** ocular diseases, development eye movement test, eye movement pattern, academic learning, eye health

## Abstract

**Objectives**: The main purpose of this work was to clinically assess the oculomotricity of one hundred Mexican children with poor reading skills but without any specific learning disorder. **Methods**: The D.E.M. psychometric test was used. Sex and age analyses of the ratio, type, horizontal and vertical performance, and errors were carried out. Results: Our data suggest that 84% of poor readers exhibit oculomotor difficulties. Sex did not significantly influence the results (*p* > 0.05), whereas age was associated with the horizontal (*p* = 0.04) and vertical (*p* = 0.29) performance, as well as the number of errors (*p* = 0.001). Omissions were the most prevalent error type. **Conclusions**: This research gives insights into the role of oculomotricity in children with poor reading skills. Our results suggest that oculomotor performance should be included in the evaluation protocol to assess poor readers to identify any influence of the visual system.

## 1. Introduction

The visual and auditory systems are the dominant sensory modalities during writing and reading. Therefore, visual–auditory processing dysfunctions could affect academic learning [1]. From the moment a child begins to stand, the visual system becomes the dominant sensory modality, as visually guided motor movements allow exploration, interaction, and learning processes to occur based on the construction of a mental representation of the world [2,3]. Research has shown that 50% of the cortex is related to the visual system, and more than thirty different cortical areas and three hundred intra- and intercortical pathways are involved in the processing of visual information [4]. Eye movements not only help in exploring the environment that surrounds us but also affect behavior and social relationships, associated with our development in the society we live in [5,6]. Hence, definition of the performance level of this system is essential when assessing children’s academic learning. In the field of neuro-optometry, the assessment of visual performance is divided into three important blocks: the sensorimotor balance of the visual system, which determines the binocular state of a child; oculomotricity, to assess spatial and temporal localization [7]; and the analysis of visual–perceptual and visual–motor skills to determine processing and motor pattern conversion abilities [8].

It has been shown that children with learning disabilities (LD) or developmental dyslexia (DD), regardless of their intellectual coefficients, present with reading and writing deficits [9]. The relationship between vision and reading has always intrigued researchers and caused great debate [10,11]. From studies on dyslexic children, it is known that neuromodulatory deficits are found to affect their visual and reading performance, and abnormalities have been associated with changes in the magnocellular and parvocellular pathway [12,13]. In general terms, slow reading performance, low comprehension, and poor writing skills are the most common symptoms [14]. Reading and writing are not innate abilities of our species and require a complex learning process that depends on the integration of multiple factors, such as saccadic eye movements, visual attention, visual–spatial and vestibular coordination, and fine motor skills and executive functions [15,16,17]. This cognitive and motor integration allows the graphical and phonetical codification [18] and contextualization and comprehension of information to occur [19]. Neuroimaging methods such as functional magnetic resonance imaging (fMRI) have provided information on the integration of diverse neural brain structures involved in reading and writing processes [20,21]. The visual cortex, cerebellum and brainstem circuitry, motor and premotor areas, and temporal and frontal lobes are some of the brain areas involved in writing and reading processes [22,23] and can be affected in children with LD and DD. Additionally, studies have shown that the inefficient magno-parvocellular coactivation can possibly be related to visual suppression and stability during reading [24], and an impaired magnocellular system can be the cause of poor temporal processing and deficient visual and auditory sequencing, which characterize DD [25]. Despite any theory behind reading difficulties, evidence has shown that efficient reading involves the coordination of the oculomotor system with cognitive processes, which impacts the brain and behavior [26].

However, what is the case for children with poor reading abilities (also referred to as reading difficulties in the absence of a specific diagnosis) who do not fall on the spectrum of LD or DD? The aim of this research was to clinically assess and classify the oculomotor patterns of children with poor reading abilities and raise awareness about the implications of these patterns for distinct aspects of the reading process [27]. Children with oculomotor deficiencies often present with spatial and temporal losses of words and lines, affecting the reading fluency and comprehension of text [28]. Oculomotricity is a term that is used in clinical practice to describe two main types of eye movement: saccades and pursuits. Saccadic movements are rapid movements of the eyes that abruptly change the point of fixation [29] and are considered to be crucial for the development of reading [30] and learning [31]. In children, a poor reading ability is defined by neuropsychologists as a score of below one standard deviation in two or more of the evaluated skills (precision, fluency, and reading comprehension) [29].

The oculomotor assessment, which explores the saccadic eye movement ability of a child, discriminates oculomotor from phonological or lexical disorders and is used to judge the reading performance and visual processing speed in children [32]. It is carried out using a psychometric test, such as the Development Eye Movement Test (D.E.M.). Previous research has shown that better reading skills are associated with increased eye movement efficiency, a factor that is primarily linked to spatial reading parameters [33].

The D.E.M. test has previously been used to evaluate dyslexic children from European [33,34,35,36] and English-speaking countries [37,38,39]. However, there has been no research on the visual efficacy and oculomotricity of Mexican children with or without specific reading disorders, making this the first study to analyze the oculomotor patterns of children with poor reading abilities in this population. Children showing signs and symptoms mostly related to some dysfunction of the visual system were referred to our department after neuropsychological assessments to rule out the presence of DD or LD. We hypothesized that an eye movement analysis using the D.E.M. test would highlight any oculomotor deficits that could impact reading skills. In Mexico, where the optometrist’s role in visual care remains unclear, children are mostly referred to an ophthalmologist for visual exams, where other key areas of visual health are checked. However, crucial elements of the visual system (sensorimotor balance, oculomotricity, and perceptual–motor skills) are left out. As a result, reading dysfunctions related to poor visual performance may go unnoticed. Through this study, we aimed to clinically determine the predominant oculomotor behavior in this group of poor readers, as the D.E.M. classification helps to differentiate oculomotor from phonological or lexical dysfunctions [32] and could serve as a guide for subsequent assessments or the determination of treatments needed. Without a comprehensive assessment, reading difficulties or poor reading abilities are mostly treated as learning problems, even without a specific neuropsychological diagnosis. This situation will persist if the root causes of such disorders are not adequately identified and treated. Looking for the cause and not the symptom should be essential to identify the appropriate treatment. Early evaluations and treatments focused on the root cause of reading difficulties promote a higher level of academic learning [40], as cortical plasticity is age related, and critical periods have the most significant impacts on the neuronal brain network [41].

This paper is structured as follows: In Section 2, the methodologies of the different analyses performed are described. In Section 3, the results are presented. In Section 4, the results are discussed. Finally, in Section 5, the conclusions are given.

## 2. Materials and Methods

In this study, the oculomotricity of children with poor reading abilities was assessed. Children were referred to us by their school’s pedagogical department as those facing reading challenges but without any specific diagnosis, such as DD or LD, that could justify their difficulties as confirmed by a previous neuropsychological assessment. The neuropsychological assessment comprises different domains, one of which determines reading abilities (fluency, precision, and comprehension). “Poor reading abilities” was the conclusion of this assessment, and children were referred to our department for further evaluation [42].

Likewise, the clinical observations gathered by the children’s teachers and neuropsychologists were mainly related to visual system dysfunction. Poor tracking skills, spatial loss during writing or reading, slow copying abilities, decreased reading fluency, head or body movements, and motor support (the need to use the index finger or a ruler during reading) were some of their concerns.

The neuro-optometric evaluation protocol included the following:

- A detailed medical history;
- The visual efficacy exam (included an assessment of ocular health to exclude pathologies or structural damages);
- Oculomotor performance;
- Visual–perceptual and visual–motor skills;
- Analysis of laterality and directionality concepts.

In this study, only the visual efficacy exam results and oculomotor performance were considered for the statistical analysis.

Data were collected at the Brain Vision & Learning Center, a research center, in collaboration with the Autonomous University of Querétaro, Mexico from June to December 2023. The study adhered to the Declaration of Helsinki guidelines, and informed consent was obtained from participants before any procedure. This study was approved by the Institutional Review Committee (CEAIFI-102-2023-TL).

Eligibility was based on the following criteria:

- Aged 6 to 13 years;
- VA ≥0.1 logMar (VA between 0.0 and 0.1 logMAR);
- No previous visual treatments;
- No ocular pathologies;
- Average IQ score for chronological age as reported by the participant’s school.

Exclusion criteria were as follows:

- Neurological conditions such as attention-deficit/hyperactivity disorder, epilepsy, depression, or any neurodevelopmental disorder;
- Use of any medication that could affect the central nervous system;
- Participants with strabismus and/or amblyopia;
- Born prematurely.

More specifically, our sample was composed by the following:

- Healthy children, without any neurological or development condition that could affect their academic performance;
- DD and LD excluded by the school’s neuropsychological department using the ENI-2 test;
- Children of similar socio-economic status (upper-middle class);
- Children from bilingual schools (private schools where English language is taught as a second language).

Clinical observations and psychometric evaluations related their poor reading abilities to a compromised visual system and not a learning problem per se, as children presented one or more of the following signs and symptoms:

- Poor tracking skills;
- Spatial loss during writing or reading;
- Slow copying abilities;
- Decreased reading fluency;
- Head or body movements;
- Motor support.

A poor reading ability is defined by the ENI-2 test “as a score of below one standard deviation in two or more of the evaluated skills (precision, fluency, and reading comprehension), while dyslexia or learning difficulties, a score of below two standard deviations”.

The age limit for participants was 13 years as suggested by the schools’ pedagogical departments, as older children generally have already compensated for their difficulties. Additionally, the target population of the D.E.M. test was children without, with, or suspected to have learning disabilities aged between 6 and 13.11 years.

Additionally, based on the related literature, a difference of 0.2 logMAR (BCVA) between the two eyes is defined as unilateral amblyopia, while a BCVA of lower than the developmental norm by ≥0.2 2 logMAR at a given age is considered bilateral amblyopia [43]. However, children with amblyopia were excluded from this study, and only those with VA scores between 0.0 and 0.1 logMAR were included.

Visual exams were performed in the morning after a good night’s sleep to avoid visual system tiredness and to ensure that reliable data were collected. Tests did not exceed 45 min and were adapted to each child.

The visual efficacy exam determines the sensorimotor balance of the visual system.

- Motor evaluations were necessary to determine the phoria state (patients with strabismus were excluded) and oculomotricity of participants.
  – The cover test was chosen to assess the motor balance of the visual system;
  – The D.E.M. test was used to assess oculomotricity.
- The sensorial evaluation assesses the degree of stereopsis, the visual acuity at both distances (to exclude patients with amblyopia), and the ocular dominance.
  – Visual acuity was measured with the Bailey–Lovie chart (logMAR) at 6 m and 40 cm;
  – Stereopsis was measured with the Random Dot 2 Test, which progresses from gross to fine stereopsis (500 to 12.5 arcminutes).

The neuro-optometric evaluation was performed with the optimal optical correction of the child.

### The Development Eye Movement Test ^TM^ (D.E.M.)

The D.E.M. test comprises three plates: two with a vertical number array (A+B) and one with a horizontal number array (C). The updated version 2.8 (2016) was used. This is distributed exclusively by the Bernell corporation.

In this test, the child is instructed to read numbers down the columns and across the rows as quickly and carefully as possible. The time needed to complete each subtest and the number of errors (omission, addition, substitution, and transposition) are recorded. The vertical subtest is mainly related to the child’s automaticity skills, while the horizontal part is related to oculomotor abilities. By dividing the adjusted horizontal time by the vertical one, the ratio is obtained. The ratio directly compares the vertical (automaticity) and horizontal (oculomotor control + automaticity) test performance results. This procedure differentiates the effect of automaticity on the oculomotor performance. However, the relationship is not always simple.

For clinical usage, four types of ratios were determined:

- Type 1: Average performance for all subtest values.
- Type 2: Characterized by an abnormally increased horizontal time in the presence of a relatively normal performance on the vertical subtest. The ratio is higher than expected. This behavior pattern is called oculomotor dysfunction.
- Type 3: Horizontal and vertical times are higher, but the ratio is average. This is related to poorly developed automaticity skills, which affect the horizontal subtest performance. This represents basic difficulties in the automaticity of number naming.
- Type 4: Vertical, horizontal, and ratio scores are abnormal. This is a combination of type 2 and 3 and indicates deficiencies in automaticity and oculomotor skills.

Additionally, Type 5 includes all cases that cannot be classified. Participants in this group show deficiencies in one or another subpart of the test, but their results cannot be classified into any of the known categories of the D.E.M. test [44].

When the test is scored, standard scores and percentile ranks are used for clinical purposes. A score of at least 1.0 standard deviation (Std) below the mean 16th percentile or a standard score (SS) of 85 are considered indicators of below-average performance. However, such assessments are controversial, which is why scores of 0.5 Std below or above are considered [31,45]. A 1.5 Std score would place the patient in the 7th percentile (78 SS), which is a problem area, whereas a 0.5 Std score would place the child below the 31st percentile (93 SS), considered to represent “at risk” or potentially problematic.

Likewise, the presence or absence of symptoms is determined to determine the normal as opposed to using abnormal standards. A 1.0 Std or 16th percentile is suggested when symptoms are not evident or reported. A 0.5 Std or 31st percentile is suggested when symptoms are present. Based on these data, our analysis focused on four different categories as follows:

- Below average performance: 16th percentile and below (from 1.0 Std below the mean—SS 85 and lower);
- At risk performance: 17th to 31st percentiles (until 0.5 Std—SS of 86 to 93);
- Approaching average performance: 32nd to 50th percentiles (SS 94–100);
- Above average performance: up to the 50th percentile.

However, for screening purposes, the ±1.0 Std scoring range was suggested as a cutoff to identify potential problems without having a significant number of over-referrals. The neuro-optometric assessment was performed by Dr. Danjela Ibrahimi, a highly qualified specialist in this field.

## 3. Results

This was a descriptive, transversal, and comparative study. A total of 100 visually normal children with a mean age of 8.0±1.8 years participated in this study, of whom 64 were male (64%) and 36 were female (36%). From the total, 86 (86%) were exophoric (mean value 8.2±4.2), and 14 (14%) were esophoric (mean value 3.6±2.4), and the mean stereopsis degree was 27.5±10.2.

Based on the statistical analysis, the results were grouped into four different categories as follows:

- Below average;
- At risk;
- Approaching average;
- Above average.

The results are divided into four sections.

### 3.1. D.E.M. Results for All Participants

#### 3.1.1. D.E.M. Type

**Figure 1:**
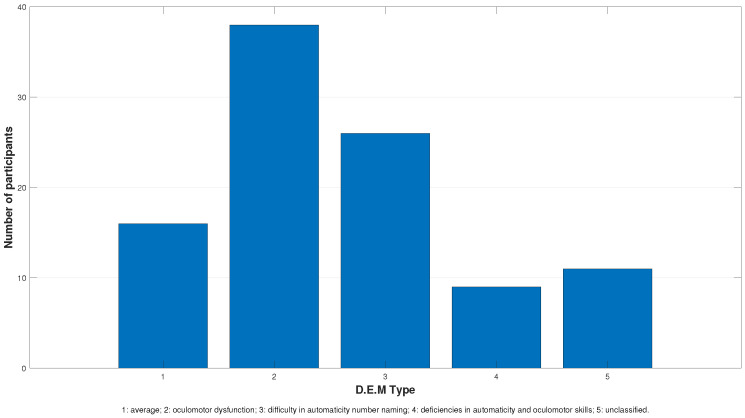
Illustrates the D.E.M. results as defined by type. The chart shows that Type 2 predominates (38%), while Type 4 is less frequent (9%). Nevertheless, the distributions of Types 4 and 5 (11%) are very similar. Type 1 makes up only 16% of the children, while Type 3 includes 26% of them.

The results from the distribution into five different categories are presented in Figure 1.

#### 3.1.2. D.E.M. Ratio

**Figure 2:**
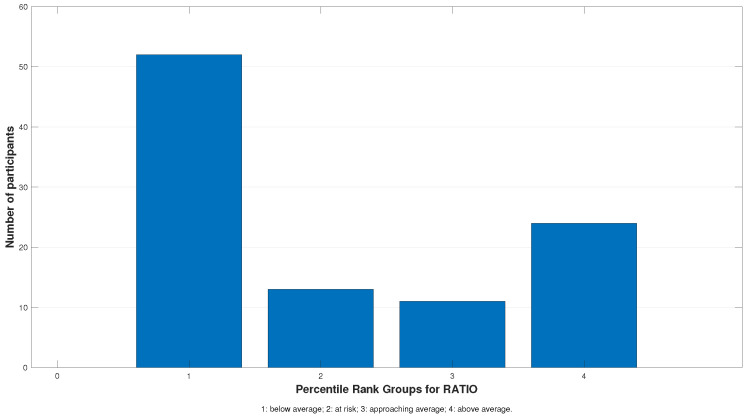
The D.E.M. ratio results grouped by percentile ranks. Group 1 represents participants with below-average performance and is predominant (52%), followed by Group 4 (24%). Similar distributions can be seen for Groups 2 (13%) and 3 (11%).

Figure 2 illustrates the D.E.M. ratio distribution based on the obtained percentile ranks.

In addition to the D.E.M. type and ratio, which are the key elements of the oculomotor psychometric test, the horizontal and vertical performance and the total number of errors were added to the statistical analysis.

The analysis of the horizontal subpart of the D.E.M. test showed that 76% of children had a below-average performance, and another 10% were classified as at risk.

A below-average performance on the vertical subtest was presented by 39% of participants, and another 20% were deemed to be at risk. For the total number of errors, 62% of children were classified as having below-average performance and 14% were classified as at risk.

### 3.2. Analysis Based on Sex

Male and female participants were compared to find similarities and/or differences in the D.E.M. test results.

To understand a child’s performance when compared to their peers without any reading difficulties, standard scores were used to analyze differences between groups by performing the independent-samples *t*-test analysis. Table 1 shows the results for the adjusted horizontal time, vertical time, ratio, and total number of errors based on sex.

Table 1 shows that the obtained SSs for the adjusted horizontal time, vertical time, ratio, and total number of errors between groups were very similar. To understand the performance of these children compared to “normal” children, an explanation of the SS is given below:

A score of at least 1.0 standard deviation (Std) below the mean, 16th percentile or a standard score (SS) of 85 is considered to indicate a below-average performance. Likewise, a score of 1.5 Std would place the patient in the 7th percentile (78 SS), which is a problem area, whereas a score of 0.5 Std would place the child below the 31st percentile (93 SS), which is considered to indicate “at risk” or potentially problematic scores. Our participants had scores of between 0.5 Std and 1 Std (16th and 31st percentile ranks), covering the range from “at risk” to “below the mean”. Therefore, they should be considered for further evaluation and treated based on their needs.

**Figure 3:**
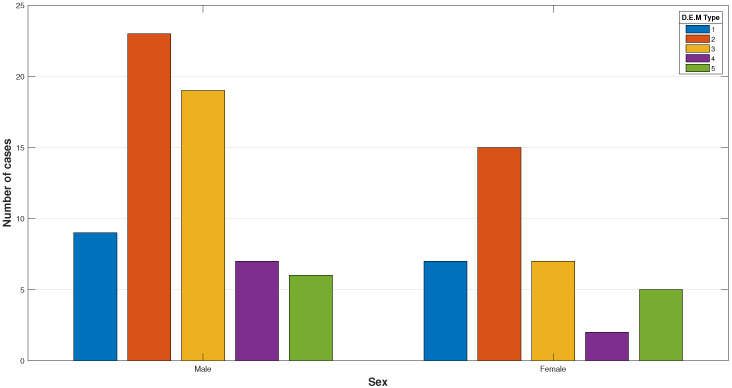
Distribution of the D.E.M. types as defined by gender. The charts show similarities between groups, with D.E.M. Type 2 being predominant, followed by Type 3.

The D.E.M. type distribution, an essential hallmark of the oculomotor performance, is presented in Figure 3.

The results suggest that oculomotor dysfunction does not depend on gender. Poor readers have the same probability of showing any type of oculomotor deficiency, independent of sex.

### 3.3. Analysis Based on Age

Patients were divided into three groups based on their reading, writing, and comprehension levels, as well as the amounts of time spent in front of digital screens and performing near-distance work.

- Group 1 (47 children): from 6.0 to 7.11 years.
- Group 2 (29 children): from 8.0 to 9.11 years.
- Group 3 (24 children): from 10.0 to 13.11 years.

To understand the effect of age on the D.E.M. results, standard score values were used to compare the horizontal and vertical performance, ratios, and total numbers of errors between age groups. The equality of variances was calculated using Levene’s test, where the *p*-value was significantly higher than 0.05. For the total number of errors, the significance was p=0.002. A one-way ANOVA with Tukey’s post hoc test for multiple comparisons was performed for the statistical analysis of the horizontal and vertical performance and ratios, and Dunnett’s post hoc test was used to assess the total number of errors. More specifically, we obtained values of F=5.381 and p=0.0006 for the horizontal performance; F=3.511 and p=0.034 for the vertical performance; F=2.331 and p=0.105 for the ratio; and F=6.412 and p=0.002 for the total number of errors.

The results showed statistically significant differences among age groups for the horizontal and vertical performance and the total number of errors. Mean values, Stds, and the *p*-values obtained in the statistical analysis are shown in Table 2.

To understand the performance of our participants when compared to their peers without any reading difficulties, we used the obtained standard score values and compared them to their percentile ranks. Table 2 shows that the lowest standard score value was 74.6 (the horizontal performance of the second group), and the highest was 91.4 (the vertical performance of the first group). As mentioned for the analysis by sex, if the obtained results lie between 0.5 and 1 Std, covering the range from “at risk” to “below the mean”, the children should be considered for further evaluation and treated according to their needs.

**Figure 4:**
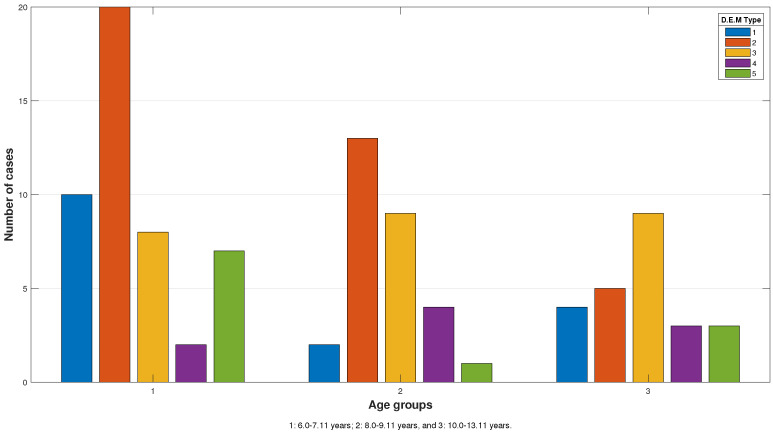
D.E.M. type distribution based on age groups. Significant differences can be seen among groups, where Type 2 predominates in participants from the first (42.6%) and second (44.8%) age groups, and Type 3 predominates in the third age group (37.5%).

The D.E.M. type distribution based on age as a key feature of the oculomotor performance is shown in Figure 4.

These data suggest that, as a child grows older, the risk of presenting with an oculomotor dysfunction decreases.

### 3.4. Analysis of the Four Types of Errors Present during the D.E.M. Test

**Figure 5:**
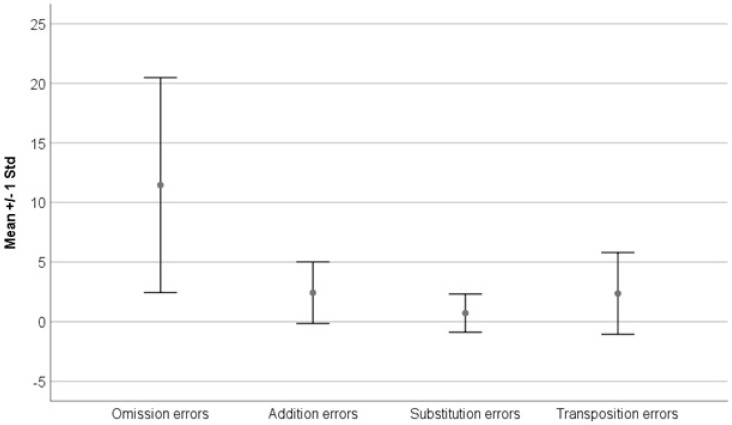
Shows the mean and Std values for the four error types presented during the D.E.M. test. Omissions (μ=11.5±9.0) were the most frequent errors, followed by additions (μ=2.4±2.6) and transpositions (μ=2.4±3.4), with substitutions being less frequent (μ=0.7±1.6).

Four different errors are included in the general D.E.M. results: omission, addition, substitution, and transposition. The mean value and standard deviation for each were calculated and are presented in Figure 5.

Types of errors (mean ± Std):

- Omission: 11.5±9.0;
- Addition: 2.4±2.6;
- Substitution: 0.7±1.6;
- Transposition: 2.4±3.4;
- Total number of errors: 16.9±11.6.

From these results, it can be deduced that these children will easily become lost when reading or copying, which can present a real challenge for their learning processes.

#### 3.4.1. Types of Errors and Sex

The analysis of each type of error based on sex was carried out using the independent-samples *t*-test, with no statistically significant differences identified between male and female participants. The mean, Std, and *p*-values for each type of error based on sex can be seen in Table 3.

#### 3.4.2. Types of Errors and Age Groups

Each error type was analyzed based on the age group. The one-way ANOVA with Dunnett’s post hoc test for multiple comparisons showed important differences between groups. The mean, Std, and *p*-values obtained in the statistical analysis are shown in Table 4.

## 4. Discussion

In this study, we measured, analyzed, and compared the oculomotor patterns of one hundred Mexican children with poor reading abilities using the psychometric D.E.M. test. In Western culture, reading is performed from left to right (horizontal visual scanning) and top to bottom (vertical visual scanning), and neurophysiology explains that vertical eye movements may be controlled by more complex systems than horizontal ones, which require precise fusion and fixation [46].

Additionally, the ability to maintain vertical control during a saccadic task could be an essential feature that differentiates oculomotor behaviors between children with below-average reading skills and those with higher reading abilities [38]. Therefore, both horizontal and vertical performance results were considered in the statistical analysis. The ratio, a variable showing the time needed to perform the horizontal and vertical subtests, was the main measure used to evaluate oculomotor patterns, as it has been demonstrated to be a predictor of oculomotor dysfunction [47,48]. Assessing children’s oculomotor performance during the reading process is more significant, as reading is integral to the learning capacity [49], and eye movements allow rapid visual scanning and tracking using saccades and fixations to extract and process visual information from a textbook [28,50]. The D.E.M. test is a useful method for determining the oculomotricity of children with and without specific learning disorders [51], dyslexia [52], brain injuries [53], reading difficulties [54], and other neurological conditions [55]. However, previous studies in this area were carried out in European or English-speaking countries. For other countries, such as China [56] and Japan [57], new normative data have been provided, showing that language is related to the test results. Nevertheless, there has been no research on the oculomotor performance of Mexican children with or without specific reading disorders.

In Mexico, there are almost 22 million children aged 5–14 years, and over 580,000 of these have a learning problem, of which 32.6% of problems are related to the visual system, 40.1% is associated with attention, concentration, and focusing difficulties, and the other 30.2% is related to language processes [58,59]. Children are mostly referred to an ophthalmologist for their annual visual exam, as the role of optometrists in visual care remains unclear, and the concept of vision and its components still needs to be explored. Therefore, crucial components of the visual system (stereopsis, oculomotricity, vergencies, accommodation, etc.) and perceptual–motor skills are not considered. As a result, reading dysfunctions related to poor visual performance may go unnoticed.

Furthermore, in the absence of a comprehensive assessment, reading difficulties are mostly treated as learning disorders, even in the absence of a specific neuropsychological diagnosis. The situation will persist if the root cause is not adequately identified and treated. Looking for the cause and not the symptom is crucial for early adequate treatment, as it is well known that age is related to cortical plasticity, and there are critical periods with significant impacts on the neuronal brain network [41].

Based on the above information, this group of children, who were referred from the school’s pedagogical department for poor reading abilities identified by the assessment of their age-referenced reading ability and who showed signs related to the functionality of the visual system (poor tracking skills, spatial loss during writing or reading, slow copying abilities, decreased reading fluency, head or body movements, or the need to use the index finger during reading or copying), underwent a detailed visual exam. The psychometric D.E.M. test was used to assess their oculomotor performance. Our results show that most participants presented with some type of oculomotor abnormality during the horizontal and/or vertical performance tests. A common characteristic among them was the high incidence of omissions during reading, which could be reflected in academic achievements. These results prove that delays in reading skills are often accompanied by oculomotricity difficulties [50], even in the absence of any specific learning disorder. Even in the absence of an eye-tracker to define the numbers of saccades, fixations, and regressions, our results relate to other studies [38,60], which have proved that below-average readers score worse in all subparts of the D.E.M. test, with the number of omissions being higher in these children.

A sex analysis was carried out to consider the structural sex-related differences across the whole brain range [61]. The statistical analysis showed that groups scored similarly for all subparts of the D.E.M. test, with the outcomes being below average in more than half of the participants. On the other hand, it has been shown that saccadic eye movements are age related [62,63] and improve as the prefrontal cortex matures. In our sample, the age analysis showed that the Type 2 ratio was more frequent in the first two groups, while Type 3 was more prevalent in the last group (the older ones), with younger children presenting more omissions, proving that, indeed, oculomotricity is dependent on the maturation of the frontal eye field and its connections with the visual cortex [64]. These data should help to raise awareness of the critical role of oculomotricity as an essential component of vision during the reading process. The oculomotor assessment should be included in the evaluation protocol when reading difficulties are suspected. This would exclude any implication of the visual system in the reading process. Unlike other recent studies that incorporated an eye-tracker for their analysis [35,38,65], we focused on showing the clinical usefulness of the D.E.M. test in the assessment of oculomotor dysfunction in children with poor reading skills.

It is important to emphasize that not all specialists caring for learning-disabled children possess an eye tracker or are qualified in its interpretation. In contrast, the D.E.M. test has no formal restrictions [44] as long as the professional has a deep understanding of the concept of vision and the cognitive status of the patient [66] and understands that a multidisciplinary approach to their difficulties is crucial to success [67]. However, a possible limit that remains is the fact that we did not use an eye tracker to compare our results with other studies. Even though the eye-tracking technology is available and affordable [68], the last version of the psychometric D.E.M. test was used, as it can be easily applied on a larger scale (even by the school’s pedagogical department) to exclude any visual system issues when reading difficulties are presented. By using the D.E.M. test for clinical purposes in poor readers with or without symptoms related to the visual system, potential problems can be identified early, and multidisciplinary treatments can prevent further academic learning delays.

The main contributions of the work are as follows:

1. This is the first study to analyze the eye movement patterns of Mexican children with poor reading abilities.
2. Through this article, we want to raise the awareness of the affect of the visual system on reading problems.
3. We show that the clinical assessment of oculomotricity using the D.E.M. test can be as powerful as using an eye-tracker to exclude eye movement dysfunctions.
4. We aim to improve the awareness that vision and visual acuity are two different concepts, and not all reading difficulties are learning disabilities; therefore, the assessment of related components should be the first step before any further neuropsychological evaluation is conducted.

Additionally, to provide reliable data, only visually normal children were included in this research. However, from the total number of patients that are referred to our department, 15% present visual dysfunctions such as strabismus, amblyopia, high refractive errors, etc. Our goal is to compare their performance in order to find differences or similarities among them. Additionally, perceptual–motor abilities will be explored, and correlations between their oculomotor patterns and perceptual–motor state will be analyzed. We aim to predict the academic performance of children with reading difficulties based on their oculomotor performance and perceptual–motor abilities, using psychometric tests and eye trackers to compare the obtained results.

## 5. Conclusions

This paper gives insights into the role of oculomotricity in 100 children with poor reading skills. Our results suggest that 84% of poor readers exhibit oculomotor difficulties, with Type 2 prevailing among others. Below-average performance was predominant for the ratio (52%), errors (62%), horizontal (76%), and vertical (39%) subparts of the D.E.M. test. Gender was not related to the results (p>0.05), whereas age was associated with the horizontal (p=0.04) and vertical (p=0.29) performance, as well as the number of errors (p=0.001). Omissions prevailed as the most common error type (11.5±9.0). Younger children had a higher rate of errors (p<0.05). These data suggest that oculomotor performance should be part of the evaluation protocol for poor readers to exclude any visual system issues.

## Figures and Tables

**Table 1:** Mean, Std, and *p*-values for male and female participants for the adjusted horizontal and vertical times, ratio, and total number of errors. The independent-samples *t*-test analysis was performed to compare means between groups. From these data, it can be appreciated that gender does not affect the oculomotor performance in this group of children.

Sex	HZ SS Mean ± Std	*p*-Value	VT SS Mean ±Std	*p*-Value	Ratio SS Mean ± Std	*p*-Value	Total Number of 0.1 Errors SS Mean ± Std	*p*-Value
Male	80.1±15.3		86.3±16.2		88.7±13.8		82±17.1	
Female	82.7±11.2	0.369	89.5±13.5	0.320	88.1±15	0.835	81.1±15.1	0.806

HZ., horizontal.; VT., vertical.; SS., standard score values.; Std, standard deviation.

**Table 2:** Mean and Std values for each variable based on the age group analysis and *p*-values for the comparisons among them. Relevant differences were only found when the first age group was compared to the second. The youngest group performed differently from the rest.

Variable	Age Group	Mean ± Std	Comparisons	*p*-Value
Horizontal SS	1	85 ± 13	1 vs. 2	0.004
	2	74.6 ± 11	1 vs. 3	0.499
	3	81.2 ± 16.4	2 vs. 3	0.181
Vertical SS	1	91.4 ± 13.1	1 vs. 2	0.029
	2	82.3 ± 14.7	1 vs. 3	0.331
	3	86.1 ± 17.8	2 vs. 3	0.627
RATIO SS	1	91 ± 13.4	1 vs. 2	0.089
	2	84 ± 14.6	1 vs. 3	0.867
	3	89.2 ± 14.6	2 vs. 3	0.365
Total Number of Errors SS	1	87.5 ± 12.8	1 vs. 2	0.001
	2	75.4 ± 13.3	1 vs. 3	0.149
	3	77.8 ± 21.8	2 vs. 3	0.951

SS, standard score values; Std, standard deviation.

**Table 3:** Mean and Std values for each type of error for male and female participants, as well as the *p*-values obtained in the independent-samples *t*-test analysis. The statistical analysis showed that sex does not affect the type or number of errors made during the D.E.M. test.

Errors	Sex	Mean ± Std	*p*-Value
Omission	Male	10.6±8.2	0.231
	Female	13.0±10.2	0.231
Addition	Male	2.2±2.5	0.166
	Female	2.9±2.6	0.166
Substitution	Male	0.6±1.3	0.351
	Female	0.9±1.9	0.351
Transposition	Male	2.5±3.5	0.611
	Female	2.1±3.3	0.611

**Table 4:** Mean and Std values for each type of error based on the age groups and the *p*-values of the comparisons among groups. The first group presented with more relevant differences than the other two, whereas the second and third groups presented with more similarities.

Errors	Age Groups	Mean ± Std	Comparisons	*p*-Value
Omission	1	15.3±8.8	1 vs. 2	0.001
	2	9.0±5.8	1 vs. 3	0.003
	3	6.9±9.7	2 vs. 3	0.722
Addition	1	3.3±2.6	1 vs. 2	0.037
	2	1.7±2.5	1 vs. 3	0.027
	3	1.7±2.1	2 vs. 3	1.000
Substitution	1	1.3±2.1	1 vs. 2	0.005
	2	0.2±.6	1 vs. 3	0.005
	3	0.2±.5	2 vs. 3	1.000
Transposition	1	3.1±3.5	1 vs. 2	0.917
	2	2.6±4.1	1 vs. 3	<0.001
	3	0.5±1.1	2 vs. 3	0.040

## Data Availability

The data presented in this study are available on request from the corresponding author due to privacy and ethical reasons.
